# Control of false discoveries in grouped hypothesis testing for eQTL data

**DOI:** 10.1186/s12859-024-05736-3

**Published:** 2024-04-11

**Authors:** Pratyaydipta Rudra, Yi-Hui Zhou, Andrew Nobel, Fred A. Wright

**Affiliations:** 1 Department of Statistics, Oklahoma State University, Stillwater, OK USA; 2 Bioinformatics Research Center, Departments of Statistics and Biological Sciences, North Carolina State University, Raleigh, NC USA; 3 Department of Statistics and Operations Research, University of North Carolina, Chapel Hill, NC USA

**Keywords:** eQTL, Grouped hypothesis testing, False discovery rate, Empirical Bayes

## Abstract

**Background:**

Expression quantitative trait locus (eQTL) analysis aims to detect the genetic variants that influence the expression of one or more genes. Gene-level eQTL testing forms a natural grouped-hypothesis testing strategy with clear biological importance. Methods to control family-wise error rate or false discovery rate for group testing have been proposed earlier, but may not be powerful or easily apply to eQTL data, for which certain structured alternatives may be defensible and may enable the researcher to avoid overly conservative approaches.

**Results:**

In an empirical Bayesian setting, we propose a new method to control the false discovery rate (FDR) for grouped hypotheses. Here, each gene forms a group, with SNPs annotated to the gene corresponding to individual hypotheses. The heterogeneity of effect sizes in different groups is considered by the introduction of a random effects component. Our method, entitled Random Effects model and testing procedure for Group-level FDR control (REG-FDR), assumes a model for alternative hypotheses for the eQTL data and controls the FDR by adaptive thresholding. As a convenient alternate approach, we also propose Z-REG-FDR, an approximate version of REG-FDR, that uses only Z-statistics of association between genotype and expression for each gene-SNP pair. The performance of Z-REG-FDR is evaluated using both simulated and real data. Simulations demonstrate that Z-REG-FDR performs similarly to REG-FDR, but with much improved computational speed.

**Conclusion:**

Our results demonstrate that the Z-REG-FDR method performs favorably compared to other methods in terms of statistical power and control of FDR. It can be of great practical use for grouped hypothesis testing for eQTL analysis or similar problems in statistical genomics due to its fast computation and ability to be fit using only summary data.

**Supplementary Information:**

The online version contains supplementary material available at 10.1186/s12859-024-05736-3.

## Background

Expression quantitative trait locus (eQTL) analysis aims to detect genetic loci that are associated with the expression of one or more genes [1]. For each gene, expression can be considered as a quantitative trait potentially associated with the genotypes at different sites in the genome, typically single nucleotide polymorphisms (SNPs) [2]. Although there is a substantial literature on both eQTL mapping [3–5] and grouped hypothesis testing [6–8], consideration of the natural gene-level grouping of the SNPs, e.g., SNPs local to a gene for a cis-eQTL problem, is comparatively unexplored or requires permutation methods or approximations [9, 10]. Analysis of gene-level eQTLs and meaningful consideration of causal SNPs is an important biological problem [11]. Testing whether there is any eQTL (local SNP) for an entire gene while controlling the false discovery rate (FDR) across the set of all genes may be interesting for various reasons, which has been imperfectly addressed in the “e-Gene” concept employed by the GTEx Consortium [12].

Local (*cis*) eQTL testing includes tests of individual SNPs nearby a gene, which leads to summaries at the gene level [12]. The natural hierarchical organization would suggest standard methods for group-level testing [6, 13]. However, local eQTL testing can include additional structure to be exploited: (i) the number of cis-eQTLs is typically large, so that explicit consideration of the proportion and “strength” of alternatives is possible; (ii) the conditional analyses of discovered eQTLs suggest that, to a first approximation, most local eQTLs can be considered unique within the region [14]; (iii) correlation of test statistics is driven by regional SNP correlation.

In the following sections, we discuss the structure of eQTL data and how the grouped nature can be effectively modeled using a random effects model. We consider the case of *cis*-eQTLs, i.e. local to the gene [14, 15], where the variant affecting the gene expression is in the immediate neighborhood of the gene. Our proposed method, entitled Random Effects model and testing procedure for Group-level FDR control (*REG-FDR*), uses an empirical Bayes framework to model the eQTL data and controls the FDR by adaptive thresholding. We also propose an alternate approach *Z-REG-FDR*, an approximate version of *REG-FDR*, that uses only the summary measures given by the Z-statistics of association between genotype and expression for each gene-SNP pair. We demonstrate using simulations and real data analysis that this approximate version performs well compared to other possible approaches while having a much faster computation time.

## Methods

### Structure of the eQTL data and the hypotheses

eQTL data can typically be expressed in the form of an expression matrix, consisting of *N* genes along with a genotype matrix which has genotypes (*m* SNPs) for the same *n* sample units. We denote the expression matrix as $Y_{N \times n}$ and the genotype submatrix corresponding to the *i*th gene as $X^{(i)}_{m_i \times n}$, $i=1,2,\ldots ,N$, where $m_i$ is the number of SNPs local to the *i*th gene. Linear modeling of eQTLs typically includes additional covariates, such as expression cofactors [12, 16]. The *t*-statistics for the partial correlations between *Y* and $X^{(i)}_{m_i \times n}$, after both are adjusted for covariates, are equivalent to the Wald statistics for the $X^{(i)}_{m_i \times n}$ when conducting the full linear regression in which *Y* is modeled as a function of $X^{(i)}_{m_i \times n}$ and the additional covariates [17, 18]. We assume that the sample size *n* is large enough that the residual degrees of freedom for the *t* statistic is sufficient to use a standard normal approximation. Thus we can can directly work with *z*-statistics for *Y* and $X^{(i)}_{m_i \times n}$, where we consider each of these matrices to have been covariate-residualized.

Let $H_{0ij}$ denote the gene-SNP level null hypothesis that there is no eQTL at the *j*th SNP local to the *i*th gene, $j=1,2,\ldots ,m_i, i=1,2,\ldots ,N$. Therefore there are $\sum _{i=1}^N{m_i}$ gene-SNP level tests. These tests can be grouped into *N* groups corresponding to the *N* genes with $m_i$ tests in the *i*th group. Define $H_{0i}$ to be the gene-level null hypothesis for the *i*th gene that there is no eQTL for the *i*th gene. Therefore the gene-level null hypothesis $H_{0i}$ can be written as

1 $$\begin{aligned} H_{0i}=\cap _{j=1}^{m_i}{H_{0ij}}, \end{aligned}$$

i.e. the gene-level null requires that all of the corresponding $m_i$ gene-SNP level hypotheses be null.

### An empirical Bayes model

We adopt an empirical Bayes approach for controlling the gene-level FDR. Empirical Bayes approaches have been used in many genetic applications, and indeed these applications have been a prime motivator for the methods [19, 20]. The advantages of using an empirical Bayes approach based on the local false discovery rate (lfdr), instead of *p*-value-based FDR-controlling approaches, has been discussed in [21] and [22]. The lfdr corresponding to the gene-level null hypothesis $H_{0i}$ is

2 $$\begin{aligned} \lambda _i(Y_i,X^{(i)}) = P(H_{0i}|Y_i,X^{(i)}), \ i=1,2,\ldots ,N. \end{aligned}$$

Here $Y_i$ denotes the *i*th row of the matrix *Y*. If we obtain the lfdr $\lambda _{i}$ for each of the gene-level hypotheses, we can control the FDR at target level $\alpha$ for gene-level testing, using the following adaptive thresholding procedure, which has been used extensively in the literature [7, 23–25].

1. Enumerate the index $i_1,i_2,\ldots ,i_N$ of the genes such that $\lambda _{i_1} \le \lambda _{i_2} \le \cdots \le \lambda _{i_N}$.
2. Reject hypotheses $H_{0i_1},\ldots ,H_{0i_L}$ where *L* is the largest integer such that
  $$\begin{aligned} \frac{1}{L}\sum _{l=1}^L{\lambda _{i_l}} \le \alpha . \end{aligned}$$

[24] and subsequently [7] showed that the adaptive thresholding procedure is valid in the sense that it controls the FDR at target level $\alpha$ for an ‘oracle’ procedure where the true parameters of the model are assumed to be known. It is asymptotically valid for a ‘data-driven’ procedure when the parameters are consistently estimated from the data. [25] proved its validity under further relaxed conditions. The proof makes use of the following result (Averaging Theorem, [19]).

Let $\text {lfdr}(z)=P(H_0|z)$ denote the lfdr for observed data *z*. Then, for a rejection region ${\mathcal {R}}$, the FDR will be given by

3 $$\begin{aligned} FDR({\mathcal {R}})=P(H_0|Z \in {\mathcal {R}})=E(\text {lfdr}(Z)|Z \in {\mathcal {R}}). \end{aligned}$$

The adaptive thresholding procedure can be used to control the FDR for testing the gene-level hypotheses $H_{0i}$’s and a similar procedure can be used to test the gene-SNP level hypotheses $H_{0ij}$’s. However, obtaining the gene-level lfdr’s is a non-trivial problem. In the next section, we propose a model which enables us to calculate the lfdr’s.

### The random effects model and testing procedure for group-level FDR control (*REG-FDR*)

Here we propose a model to obtain the gene-level lfdr values, that can be subsequently used to test the gene-level hypotheses while controlling the FDR using the adaptive thresholding method. The model is based on the following assumptions.

1. For any gene *i*, under the gene-level alternative hypothesis $H_{0i}^c$, there exists a single causal SNP that influences its expression.
2. Each of the $m_i$ SNPs has equal probability to be the causal SNP.

First, we note that Assumption (A1) is at best a simplification, but very large eQTL studies have supported the view that most genes with eQTLs have a primary local eQTL [26], with other loci having much smaller effect sizes. We therefore treat A1 as a ‘workable condition’ [27–29].

Assumption (A2) could easily be relaxed, and one might use a distributional assumption over the SNPs as a modest modification of our method below (see the Discussion section). We note that it is trivial to enforce Assumption (A2) by, for example, randomizing the SNP identities within gene *i* prior to analysis.

Under these assumptions, the gene-level lfdr for the *i*th gene has the following form:

4 $$\begin{aligned} \lambda _i(Y_i,X^{(i)})= P(H_{0i}|Y_i,X^{(i)})&= \frac{P(H_{0i})P(Y_i,X^{(i)}|H_{0i})}{P(H_{0i})P(Y_i,X^{(i)}|H_{0i}) + P(H_{1i})P(Y_i,X^{(i)}|H_{1i})} \end{aligned}$$

5 $$\begin{aligned}&= \frac{\pi _0f_0(Y_i)}{\pi _0f_0(Y_i)+(1-\pi _0)\frac{1}{m_i}\sum _{j=1}^{m_i}{f_1(Y_i|X_j^{(i)},\beta _{ij})}}, \end{aligned}$$

where $\pi _0=P(H_{0i})$ is the prior probability of $H_{0i}$, $f_0(Y_i)$ is the density of $Y_i$ under the null, and $f_1(Y_i|X_j^{(i)},\beta _{ij})$ is the conditional density under the alternative given that the *j*th SNP is causal. Here $\beta _{ij}$ is correlation between the expression of the *i*th gene and the causal SNP *j*. Note that the marginal density $p(X^{(i)})$ cancels from numerator and denominator. Importantly, this cancellation allows us to bypass the modeling of the dependence structure of the SNPs, which otherwise might have been difficult to estimate accurately.

We assume that $f_0(.)$ is the density of the $N_n(0,I_n)$ distribution (noting that expression data can be normalized), and that $f_1(.|X_j^{(i)},\beta _{ij})$ is the density of the $N_n(\beta _{ij} X_j^{(i)}, (1-\beta _{ij}^2)I_n)$ distribution, where $\beta _{ij}$ is the correlation between $Y_i$ and $X_j^{(i)}$. This choice of $f_1$ ensures that the unconditional variance of $Y_i$ is free of $\beta _{ij}$. To account for variability across genes, we assume $\beta _{ij}$ to be a random effect such that $\sqrt{n-3} \ tanh^{-1}(\beta _{ij})$ follows a $N(0,\sigma ^2)$ distribution. As $\beta _{ij}$ is a correlation coefficient, the Fisher transformation is used to ensure that the variance does not depend on the mean. Moreover, $\sigma$ will be estimated from the data, and so the apparent dependence on *n* is not important to the procedure.

Our procedure treats the genotype values as fixed, and assuming the expression of genes to be independent, given genotypes, we can estimate $\pi _0$ and $\sigma$ using a maximum likelihood approach and follow with plug-in estimates to obtain estimates of $\lambda _i(Y_i,X^{(i)})$ from Eq. 5. The assumption that the expression of different genes are independent is violated in general, but our approach can be viewed as employing a composite likelihood [30], and thus consistent for $\pi _0$ and $\sigma$ even under independence violations [31]. An EM algorithm is used (see Additional file 1: Section 1) for the maximum likelihood estimation. The procedure enables us to use the adaptive thresholding procedure to provide proper gene-level control of the FDR.

### The *Z-REG-FDR* model

One computational challenge presented by the *REG-FDR* model is that the density $f_1(Y_i|X_j^{(i)})$ does not have a closed form expression. While it can be expressed as the following integral

6 $$\begin{aligned} f_1(Y_i|X_j^{(i)})=\int _{-1}^{1}{f_1(Y_i|X_j^{(i)},\beta ) \frac{\sqrt{n-3}}{\sqrt{2\pi }\sigma (1-\beta ^2)}e^{-\frac{n-3}{2\sigma ^2}\{tanh^{-1}(\beta )\}^2} d\beta ~,} \end{aligned}$$

numeric maximum likelihood estimation is computationally burdensome. We propose an alternative model, termed *Z-REG-FDR*, which avoids this problem. In this approach, we consider the Fisher transformed and scaled *z*-statistics as our data. Thus, for each gene *i*, we have a vector of *z*-statistics

$$z^{(i)} = (z_{1}^{(i)},z_{2}^{(i)},\ldots ,z_{m_i}^{(i)}) , \ i=1,2,\ldots ,N,$$

where $z_{j}^{(i)}=\sqrt{n-3} \ tanh^{-1}(r_{j}^{(i)})$ and $r_{j}^{(i)}$ is the sample correlation of $Y_i$ and $X_j^{(i)}$.

Fisher transformation and scaling ensures that $z^{(i)}$ is approximately normal and that the variance of each component is approximately 1 under both null and alternative. Under the null, the mean of $z^{(i)}$ is zero. We treat the component $z^{(i)}$ as if they are independent across different genes, again relying on approximate conditional independence (given genotypes) and a compositie likelihood interpretation.

The *Z-REG-FDR* procedure is based on an additional assumption to (A1) and (A2) above. If the *k*th SNP is causal, we assume (Assumption (A3)) that the distribution of $(z_{1}^{(i)},\ldots ,z_{k-1}^{(i)},z_{k+1}^{(i)},\ldots ,z_{m_i}^{(i)})$ given $z_{k}^{(i)}$ under the alternative is same as that under the null. In particular, we note that this assumption is true if the components of $z^{(i)}$ have a Markov dependence structure with the same serial correlation under null and alternative, which is true in the special case that the successive marker correlations are zero. In general, this assumption can be violated, but as shown in “Simulations: performance of *Z-REG-FDR* as an approximate maximum likelihood estimation” section, the resultant procedure appears to work well in many circumstances as an approximate maximum likelihood method even when Assumption (A3) is not satisfied.

Under the above assumptions, we can write the joint distribution of the random vector $z^{(i)}=(z_{1}^{(i)},z_{2}^{(i)},\ldots ,z_{m_i}^{(i)})$ as

7 $$\begin{aligned} f_0(z_{1}^{(i)},z_{2}^{(i)},\ldots ,z_{m_i}^{(i)})=p_0(z_{k}^{(i)})f_{0|k}(z_{1}^{(i)},\ldots ,z_{k-1}^{(i)},z_{k+1}^{(i)},\ldots ,z_{m_i}^{(i)}) \end{aligned}$$

under the null, and

8 $$\begin{aligned} f_1(z_{1}^{(i)},z_{2}^{(i)},\ldots ,z_{m_i}^{(i)})=p_1(z_{k}^{(i)})f_{0|k}(z_{1}^{(i)},\ldots ,z_{k-1}^{(i)},z_{k+1}^{(i)},\ldots ,z_{m_i}^{(i)}) \end{aligned}$$

under the alternative. We assume $p_0(.)$ to be *N*(0, 1) and $p_1(.)$ to be $N(\mu ,1)$, where $\mu$ is assumed to be random with a $N(0,\sigma ^2)$ distribution. We do not assume anything about the form of $f_{0|k}$ except that it does not involve the parameters $\pi _0$ and $\sigma$. Under these assumptions, the gene-level lfdr for this model reduces to

9 $$\begin{aligned} P(H_{0i}|z^{(i)})=\frac{1}{1+\frac{1-\pi _0}{\pi _0} \frac{1}{m_i} \sum _{k=1}^{m_i}{\frac{p_1(z_{k}^{(i)})}{p_0(z_{k}^{(i)})}}}, \ i=1,2,\ldots ,N. \end{aligned}$$

This follows from the cancellation of $f_{0|k}(z_{1}^{(i)},\ldots ,z_{k-1}^{(i)},z_{k+1}^{(i)},\ldots ,z_{m_i}^{(i)})$ in the numerator and denominator. While estimating $\pi _0$ and $\sigma$, a similar cancellation helps us bypass maximizing the full (approximate) likelihood

$$\begin{aligned}\prod _{i=1}^N{(\pi _0 f_0(z^{(i)})+(1-\pi _0)f_1(z^{(i)}))}.\end{aligned}$$

Instead, we maximize

$$\begin{aligned}\prod _{i=1}^N{\frac{\pi _0 f_0(z^{(i)})+(1-\pi _0)f_1(z^{(i)})}{f_0(z^{(i)})}} = \prod _{i=1}^N\left\{ \pi _0+(1-\pi _0) \frac{1}{m_i} \sum _{k=1}^{m_i}{\frac{p_1(z_{k}^{(i)})}{p_0(z_{k}^{(i)})}}\right\} .\end{aligned}$$

This is equivalent to the maximum likelihood estimation under the assumption that $f_{0|k}$ does not involve the parameters $\pi _0$ and $\sigma$. Note that we need to estimate only the parameters $\pi _0$ and $\sigma$ to obtain the gene-level lfdr using Eq. 9.

When the required assumptions are not satisfied, this method still has value as an approximate maximum likelihood approach. For instance, when the $X_j^{(i)}$’s are related by an AR(1) structure, it can be shown that the correlation between the *z*-statistics depends on the effect size, i.e. the correlation between $Y_i$ and the causal SNP, hence violating Assumption (A3). Additional file 1: Lemma 1 and Additional file 1: Figures 1 and 2 show the extent to which the conditional distribution $f_{0|k}$ might depend on the effect size for any correlation structure among normally distributed SNPs. However, our results in “Simulations: performance of *Z-REG-FDR* as an approximate maximum likelihood estimation” section demonstrate that it does not have a significant adverse effect on the performance of the estimation and control of false discovery.

## Results

### Simulations: performance of *Z-REG-FDR* when all assumptions are satisfied

First, we conducted a simulation study to explore the performance of *Z-REG-FDR* under the ideal situation where all assumptions are satisfied. Table 1 shows the results for simulated datasets (1000 simulations of datasets with 10,000 genes and 200 samples) where *z*’s are directly simulated from an autoregressive structure, and therefore Assumption (A3) is also satisfied. The estimates are accurate to within about 15% when the true $\sigma$ is at least 2.0. The control of the FDR is also satisfactory for $\sigma > 2$. However, the performance is not as good for small $\sigma$, which is due to the fact that it is difficult to separate the null and alternative cases when the effect sizes are small; this is true even when all the assumptions are satisfied. This is a property of the two group mixture model in the empirical Bayes set up, and not a limitation due to the approximate nature of *Z-REG-FDR*.

**Table 1:** Summary of the simulation studies with directly simulated *z* from an AR(1) model with correlation $\rho$

True $\pi _0$	True $\sigma$	True $\rho$	Mean $\hat{\pi _0}$	Mean ${\hat{\sigma }}$	SE($\hat{\pi _0}$)	SE(${\hat{\sigma }}$)	Realized	Realized
							FDR (5%)	FDR (10%)
0.20	1	0.10	0.2030	0.9964	0.1841	0.0823	0.0954	0.1236
0.20	2	0.10	0.1865	1.9660	0.0469	0.0374	0.0576	0.1136
0.20	5	0.10	0.1977	4.9383	0.0094	0.0306	0.0507	0.1014
0.20	1	0.50	0.1932	0.9919	0.1613	0.0757	0.0922	0.1252
0.20	2	0.50	0.1873	1.9663	0.0417	0.0352	0.0565	0.1121
0.20	5	0.50	0.1977	4.9383	0.0092	0.0303	0.0508	0.1013
0.20	1	0.80	0.1857	0.9875	0.1308	0.0664	0.0882	0.1245
0.20	2	0.80	0.1894	1.9673	0.0325	0.0317	0.0545	0.1090
0.20	5	0.80	0.1979	4.9388	0.0085	0.0292	0.0507	0.1012

The last two columns show the FDR control performance of the Z-REG-FDR method when the target FDR is 5% and 10%, respectively

### Simulations: performance of *Z-REG-FDR* as an approximate maximum likelihood estimation

We wished to study the accuracy of the estimation under the approximations employed and for a relatively small sample size, in order to ensure that the approach can work in this challenging situation. Accordingly, we simulated data that uses the covariate adjusted genotype matrix of a real dataset from the GTEx project (V3) [12]. The genotype matrix corresponding to the tissue ‘heart’, which had 83 samples, was selected for analysis. For computational purposes, 10,000 genes were chosen randomly from 28,991 genes. Use of genotype matrices from real data ensures that we are not enforcing Assumption (A3) while simulating, and our choice of $f_{0|k}$ for the simulation is obtained from the data. We simulate the $Y_i$’s (1,000 simulations) using the following scheme.

1. For each gene, decide whether it has an eQTL using a Bernoulli($\pi _0$) distribution.
2. If the gene has an eQTL, pick a causal SNP using a discrete uniform distribution over the $m_i$ SNPs. Let it be the *k*th SNP.
3. If the gene has an eQTL, simulate each element of $Y_i$ from $N(\beta _{ij} X_k^{(i)}, 1-\beta _{ij}^2)$ with $\sqrt{n-3} \ tanh^{-1}(\beta _{ij})$ simulated from a $N(0,\sigma ^2)$ distribution. If the gene doesn’t have an eQTL, simulate each element of $Y_i$ from *N*(0, 1).

Table 2 shows the results for this data, indicating that the estimates are still accurate and control of FDR is satisfactory unless $\sigma$ is very small. Large eQTL studies have observed large effect sizes for cis-eQTL analysis [15, 32] which implies that $\sigma$ is not expected to be very small. Thus our numerical results indicate that the *Z-REG-FDR* method has valid applications for eQTL data.

**Table 2:** Summary of the simulation studies using the SNP matrix from real data. The last two columns show the FDR control performance of the Z-REG-FDR method when the target FDR is 5% and 10%, respectively

True $\pi _0$	True $\sigma$	Mean $\hat{\pi _0}$	Mean ${\hat{\sigma }}$	SE($\hat{\pi _0}$)	SE(${\hat{\sigma }}$)	Realized	Realized
						FDR (5%)	FDR (10%)
0.10	1	0.1665	1.0771	0.0829	0.0479	0.0415	0.0659
0.10	2	0.0871	2.0443	0.0234	0.0234	0.0616	0.0964
0.10	5	0.0994	5.1088	0.0073	0.0221	0.0509	0.0974
0.20	1	0.2599	1.0802	0.0846	0.0534	0.0512	0.0903
0.20	2	0.1864	2.0437	0.0237	0.0263	0.0568	0.1106
0.20	5	0.1986	5.1075	0.0080	0.0275	0.0518	0.1017

**Fig. 1:**
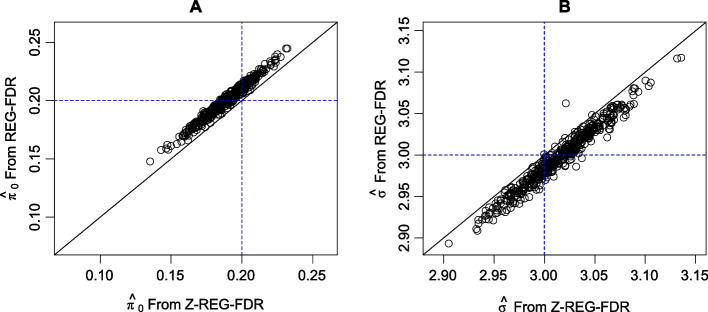
Comparison of the parameter estimates using *REG-FDR* and *Z-REG-FDR*. Except a small number of cases, the two estimates agree with each other. The blue lines show the true values of the parameters

**Fig. 2:**
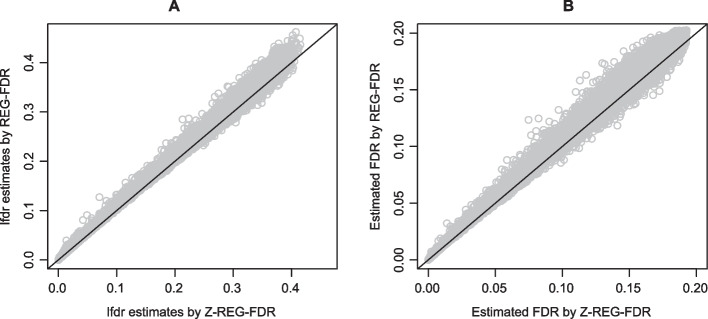
**A.** Estimated lfdr and **B.** estimated FDR for *REG-FDR* and *Z-REG-FDR*

Figure 1 shows the plot of *REG-FDR* estimates against the *Z-REG-FDR* estimates for 500 simulated datasets using the simulation scheme described above. It is clear from the plot that the two methods agree with each other (with correlations 0.906 and 0.952 for $\pi _0$ and $\sigma$, respectively) and largely fall near the unit line. These results suggest that the approximate maximum likelihood method in *Z-REG-FDR* is quite effective in controlling the FDR, with a much improved computation speed—a few minutes on a single computer for a dataset with 10,000 genes and 100–200 samples as opposed to more than a day for *REG-FDR*. A comparison of the estimated lfdr and estimated FDR of the two methods is shown in Fig. 2. It is evident that the slight over-estimation of $\pi _0$ and the slight underestimation of $\sigma$ by *Z-REG-FDR* work in opposite directions, which leads to similar lfdr values when compared to *REG-FDR*. The correlation between the estimated FDR based on the true values of the parameters and that based on *REG-FDR* or *Z-REG-FDR* are also very high (see Additional file 1: Figure 3).

### Behavior of the expected pseudo-log-likelihood of *Z-REG-FDR*

**Fig. 3:**
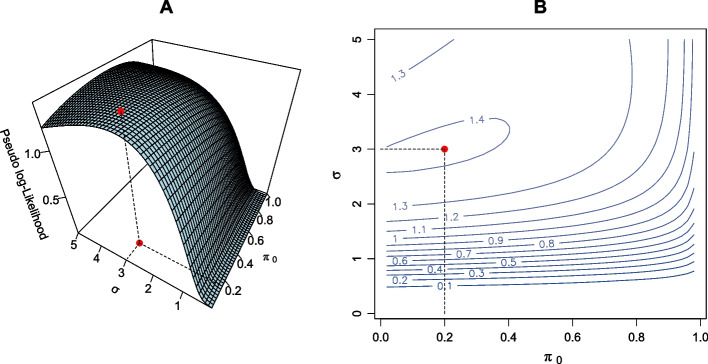
Demonstration of the optimization of log-likelihood properties using *Z-REG-FDR* method. **A.** Surface plot and **B.** Contour plot of expected pseudo-log-likelihood surface for the *Z-REG-FDR* method. True $\pi _0$ and $\sigma$ are 0.2 and 3 respectively

It is a standard result that the expected log-likelihood is maximized at the true value of the parameter under standard regularity conditions [33]. Since *REG-FDR* is the true maximum likelihood method for the proposed model, it is expected to satisfy this property. If Assumption (A3) is not satisfied then *Z-REG-FDR* is an approximate maximum likelihood method, and as such, its pseudo-log-likelihood need not be maximized at the true value of the parameter. We explored several realistic combinations of the true parameters and observed that the pseudo-log-likelihood of *Z-REG-FDR* is maximized very near the true parameter value. It is a difficult task to analytically compute the expected pseudo-log-likelihood, and so Monte-Carlo integration was used for this task. Figure 3 shows the expected pseudo-log-likelihood surface of *Z-REG-FDR* for $\pi _0=0.2$ and $\sigma =3$. A contour plot also confirms the fact the surface peaks near the true values of the parameters.

### Simulations: comparison of *Z-REG-FDR* with other methods

It is possible to use other methodologies to control the FDR in grouped hypothesis testing problem for eQTL data. A conservative approach is to obtain the Bonferroni adjusted *p*-values for each gene, where the *p*-value for each gene-SNP pair is computed based on the usual *t*-test or *z*-test, and then use an FDR controlling approach [eg 34, 35, 36] to assess the conservative *p*-values. [29] used a permutation based (“eGene”) approach in their analysis of the GTEx data. The method uses the smallest gene-SNP *p*-value for a gene as the test statistic and computes its distribution by permuting the expression values. Such a distribution can be used to obtain *p*-values for each gene, which can subsequently be used to control the FDR by using methods such as Storey’s q-value method [35].

**Fig. 4:**
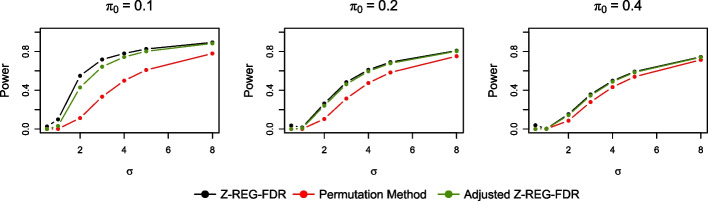
Power curves of different methods for varying combinations of the true parameter values

The Bonferroni method is typically conservative and hence less powerful. The permutation method, while correctly controlling false positives, can suffer from lack of power to detect genes having an eQTL since it uses an extreme value statistic (not based on likelihood). Our model, on the other hand, utilizes more information through its use of approximate likelihood. We carried out a simulation study to compare the performance of the methods in terms of their power. The simulations were performed using the simulation scheme described in “Simulations: performance of *Z-REG-FDR* as an approximate maximum likelihood estimation” section and statistical power was obtained using an FDR threshold of 0.05. The results are shown in Fig. 4. As expected, the Bonferroni method turned out to have very low power and is not shown in Fig. 4. The permutation approach with Storey’s *q*-value method [35] was conservative and less powerful in comparison with *Z-REG-FDR*. To address the possible concern that *Z-REG-FDR* can be slightly anti-conservative, and therefore the comparison with the permutation method is unfair, we also included an adjusted version of the *Z-REG-FDR* method where a slightly lower FDR threshold was chosen based on the simulations in such a way that the estimated FDR was exactly 0.05. This adjusted version had slightly less power compared to unadjusted *Z-REG-FDR*, but was more powerful than the permutation method.

### Analysis of real data

Finally, we also applied the *Z-REG-FDR* on a real dataset obtained from GTEx (V6) [12]. Besides *Z-REG-FDR*, we also used the permutation method and Simes method [37], which is expected to be more powerful than the Bonferroni method although it may not control the FDR for all types of correlation structures. We applied each method on the GTEx data for 44 tissues, separately for each tissue.

For each tissue, the normalized gene expression data and SNP genotype data were separately residualized after adjusting for covariates provided by GTEx. We fit a linear regression model with individuals’ gene expression or SNP genotype as the response variable and covariates as the explanatory variables. Then we extracted the model residuals to obtain “covariate-corrected” gene expression and SNP genotypes.

**Fig. 5:**
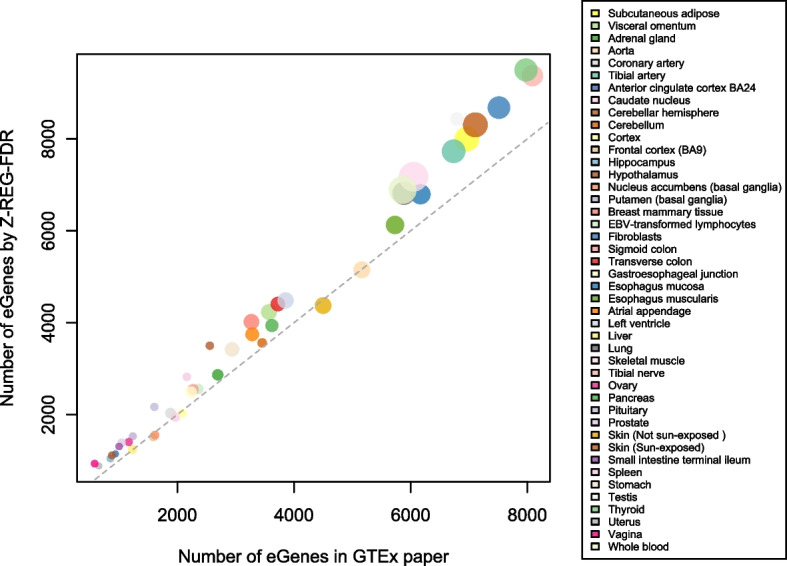
Comparison of *Z-REG-FDR* and the permutation method for GTEx data

**Fig. 6:**
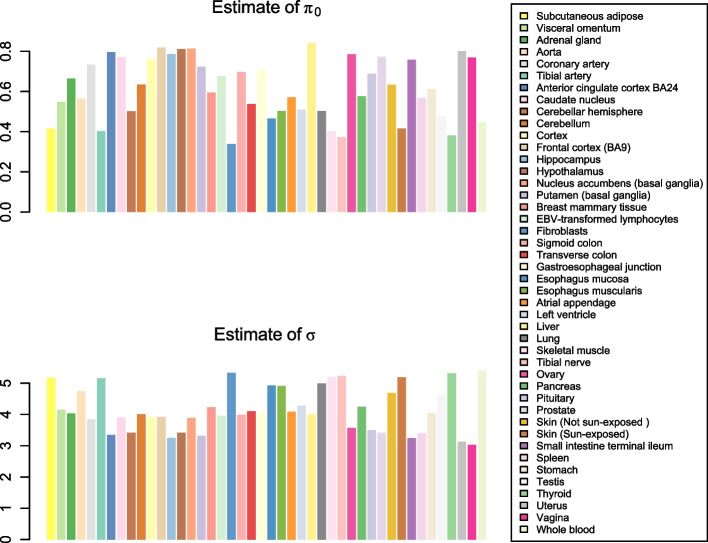
Parameter estimates using *Z-REG-FDR* for the GTEx data

Figure 5 shows a comparison of the number of significant genes found by *Z-REG-FDR* and the permutation method employed by [12]. A complete list of the sample sizes and the number of significant genes discovered for the 44 tissues is provided in Additional file 1: Table 2. The methods agree with each other to some extent in terms of number of discoveries. The *Z-REG-FDR* method has higher number of discoveries compared to the Permutation method and the Simes method in most cases. The parameter estimates for each tissue using *Z-REG-FDR* are shown in Fig. 6.

## Discussion

We have introduced a principled procedure to perform gene-level FDR test, most appropriate and useful in the eQTL setting. A major advantage of *Z-REG-FDR* is its computational efficiency. While other methods such as the permutation method or our *REG-FDR* method can take days on a single PC to complete the analysis of a real eQTL dataset, *Z-REG-FDR* can do the same in a few minutes. For instance, it takes approximately two minutes to fit the model and find significant genes by *Z-REG-FDR* for a data set with 4.5 million SNPs grouped as local SNPs for 10, 000 genes. *REG-FDR* takes about a day, and the permutation method (for 10, 000 permutations) takes about 6 hours to analyze the same data. Since there are thousands of simultaneous tests, even 10, 000 permutations may not be enough to provide sufficient *p*-value resolution. While the Bonferroni method is very fast, it has little power to detect the genes having true eQTLs.

*Z-REG-FDR* has additional advantages. One important feature of the method is that it does not require access to the full data. In fact, the symmetry of the distributions involved in the *Z-REG-FDR* pseudo-likelihood ensure that only the gene-SNP level *p*-values (or equivalently the absolute *z*-values) are needed to fit the model. *Z-REG-FDR* does not model the correlation structure of the SNPs, and therefore does not require access to that data. This might be very useful since, in many genetic applications, data are found in the form of summary measures.

*Z-REG-FDR* can be slightly anti-conservative depending on the true values of the parameters. Various simulations show that if $\sigma$ is large, which appears to often be the case for eQTL data, the control of FDR is satisfactory. The fact that Assumption (A3) is not satisfied does not significantly affect the FDR control. Therefore the assumption can be thought of as a means to reduce computational burden, rather than a necessary assumption for the practical workability of the model.

Assumptions (A1) and (A2) also have the potential to be relaxed, although we consider that to be beyond the scope of this paper. For example, the method can be extended by relaxing Assumption (A2) and incorporating a non-uniform prior for the causal location. If a well-grounded prior exists, then it can be incorporated into our method in a straightforward manner using weighted versions of our statistics. We have included an example in the Additional file 1 to demonstrate empirical evidence that the method remains valid even for more than one causal SNPs under certain conditions.

Our use of the lfdr statistics, while valid, does not utilize gene-level local correlation structures [38–41] that might provide additional power. Implementation of such methods would require sensitive estimation of gene-level correlations, and a possible direction of future effort.

With the continuous increase in the size of genomic data sets, and with the possibility of further extensions of our approach, we strongly believe that the approximate likelihood approach of the *Z-REG-FDR* method can be of great practical use for grouped hypothesis testing for eQTL analysis or similar problems in statistical genomics.

### Supplementary Information

**Additional file 1:** Supplementary Materials.

## Data Availability

Supplementary material is available in the file Supplementary.pdf. Software in the form of R code and documentation is available at https://doi.org/10.5281/zenodo.8331734.
