# Supplementary material for: Control of false discoveries in grouped hypothesis testing for eQTL data
Source: BMC Bioinformatics. 2024 Apr 11;25:147. doi: 10.1186/s12859-024-05736-3 (PMC11007981; doi:10.1186/s12859-024-05736-3)
Supplement: Supplementary file 1 — Additional file 1: Supplementary Materials. [file 12859_2024_5736_MOESM1_ESM.pdf]

Control of False Discoveries in Grouped Hypothesis Testing for eQTL Data
Supplementary Materials

Pratyaydipta Rudra[1, *], Yi-Hui Zhou[2], Andrew Nobel[3], and Fred A. Wright[4]

[1]Department of Statistics, Oklahoma State University, Stillwater, OK, USA
[2,4]Bioinformatics Research Center, Departments of Biological Sciences and Statistics, North Carolina State University, Raleigh, NC, USA
[3]Department of Statistics and Operations Research, University of North Carolina, Chapel Hill, NC, USA
[*]Corresponding author. Email: prudra@okstate.edu

1 An EM algorithm to estimate *REG-FDR* parameters

The log-likelihood for *REG-FDR* is

$$L(\pi_0, \sigma|X, Y) = log(p(X)) + \sum_{i=1}^{N} log[\pi_0 f_0(Y_i) + (1-\pi_0)\frac{1}{m_i}\sum_{j=1}^{m_i} f_1(Y_i|X_j^{(i)}, \sigma)]$$

where $p(X)$ is the marginal density of X that we avoid modelling, but assume to be free of π_0 and σ. We introduce the following unobserved variables.

> $\delta_i = 1$ or 0 according as the ith gene has an eQTL or not, $i = 1, 2, ..., N$.
> $S_{ij} = 1$ or 0 according as the jth SNP local to the ith gene is causal or not, $j = 1, 2, ..., m_i$.

Given the data (X, Y), δ_i follows $Bernoulli(1-\pi_0)$. Given the data and $\delta_i = 1$, $(S_{i1}, S_{i2}, ..., S_{im_i})$ follows a $Multinomial(1; 1/m_i, 1/m_i, ..., 1/m_i)$ distribution.
Now the complete log-likelihood becomes

$$L_c(\pi_0, \sigma|X, Y, \delta, S)$$
$$= \log(p(X)) + \sum_{i=1}^{N} \log[(\pi_0 f_0(Y_i))^{(1-\delta_i)}((1-\pi_0)\tfrac{1}{m_i}\prod_{j=1}^{m_i} f_1(Y_i|X_j^{(i)}, \sigma)^{S_{ij}})^{\delta_i}]$$

$= \log(p(X)) + \sum_{i=1}^{N} (1-\delta_i)\log(f(Y_i))$
$+ \sum_{i=1}^{N} [(1-\delta_i)\log(\pi_0) + \delta_i \log(1-\pi_0)] + \sum_{i=1}^{N}\sum_{j=1}^{m} S_{ij}\delta_i log[f_1(Y_i|X_j^{(i)}, \sigma)]$

The M-step gives

$$\hat{\pi_0} = \frac{1}{N}\sum_{i=1}^{N}(1-\delta_i)$$

and

$$\hat{\sigma} = \underset{\sigma}{ArgMax} \sum_{i=1}^{N}\sum_{j=1}^{m} S_{ij}\delta_i \log[f_1(Y_i|X_j^{(i)}, \sigma)]$$

In the kth iteration, the E-step replaces δ_i by $E(\delta_i|X, Y, \hat{\pi}_0^{(k-1)}, \hat{\sigma}^{(k-1)})$ and $S_{ij}\delta_i$ by $E(S_{ij}\delta_i|X, Y, \hat{\pi}_0^{(k-1)}, \hat{\sigma}^{(k-1)})$. These are given by

$$E(\delta_i|X, Y, \hat{\pi}_0^{(k-1)}, \hat{\sigma}^{(k-1)}) = \frac{(1-\hat{\pi_0}^{(k-1)})\frac{1}{m_i}\sum_{j=1}^{m_i} f_1(Y_i|X_j^{(i)}, \hat{\sigma}^{(k-1)})}{\hat{\pi_0}^{(k-1)} f_0(Y_i) + (1-\hat{\pi_0}^{(k-1)})\frac{1}{m_i}\sum_{j=1}^{m_i} f_1(Y_i|X_j^{(i)}, \hat{\sigma}^{(k-1)})}$$

and

$$E(S_{ij}\delta_i|X, Y, \hat{\pi}_0^{(k-1)}, \hat{\sigma}^{(k-1)}) = E(\delta_i|X, Y, \hat{\pi}_0^{(k-1)}, \hat{\sigma}^{(k-1)}) \times \frac{f_1(Y_i|X_j^{(i)}, \hat{\sigma}^{(k-1)})}{\sum_{t=1}^{m_i} f_1(Y_i|X_t^{(i)}, \hat{\sigma}^{(k-1)})}$$

The updating continues until $|L(\hat{\pi}_0^{(k+1)}, \hat{\sigma}^{(k+1)}|X, Y) - L(\hat{\pi}_0^{(k)}, \hat{\sigma}^{(k)}|X, Y)|$ becomes sufficiently small.

2 Dependence of conditional distribution on the correlation structure

The following lemma shows the extent to which the conditional distribution $f_{0|k}$ might depend on the effect size for any correlation structure among normally distributed SNPs. We use a trivariate normal distribution for illustration, as it is rich enough for demonstration while still analytically tractable.

Lemma 1. *Suppose* (X_1, X_2, X_3) *are jointly normal with mean* $(0, 0, 0)$ *and covariance ma-*

trix

$$\begin{pmatrix} 1 & \rho_1 & \rho_2 \\ \rho_1 & 1 & \rho_3 \\ \rho_2 & \rho_3 & 1 \end{pmatrix}.$$

Let $Y = \beta X_1 + \epsilon$, *where* $\epsilon \sim N(0, 1-\beta^2)$, *and* r_1, r_2, r_3 *denote the sample product moment correlation coefficient of* Y *with* X_1, X_2 *and* X_3 *respectively for a sample of size* n. *The asymptotic correlations between these sample correlations are given by*

$$Cor(r_1, r_2) = \rho_{12} = \frac{\rho_1(2 - \beta^2 - \beta^2\rho_1^2)}{2n(1-\beta^2\rho_1^2)}$$

and

$$Cor(r_2, r_3) = \rho_{23} = \frac{2\rho 3 + \beta^2(\rho_1^2 + \rho_2^2)(\beta^2\rho_1\rho_2 - 2\rho 3) + \beta^2\rho_1\rho_2(\rho_3^2 - 1)}{2n(1-\beta^2\rho_1^2)(1-\beta^2\rho_2^2)},$$

ρ_{13} *having the same form as* ρ_{12}.

Proof. For the ith sample, let us define

$$\mathbf{Z}_i = (X_{1i}, X_{2i}, X_{3i}, Y_i, X_{1i}^2, X_{2i}^2, X_{3i}^2, Y_i^2, X_{1i}Y_i, X_{2i}Y_i, X_{3i}Y_i).$$

Clearly, $E(\mathbf{Z}_i) = \mu = (0,0,0,0,1,1,1,1,\rho_1,\rho_2,\rho_3)$, and suppose $V(\mathbf{Z}_i) = \Sigma = (\sigma_{ij})_{11\times 11}$.

Define the functions g_1, g_2 and g_3, all $\mathbb{R}^{11} \to \mathbb{R}$, as

$$g_1(x_1, x_2, ..., x_{11}) = \frac{x_9 - x_1x_4}{\sqrt{(x_5 - x_1^2)(x_8 - x_4^2)}},$$

$$g_2(x_1, x_2, ..., x_{11}) = \frac{x_{10} - x_2x_4}{\sqrt{(x_6 - x_2^2)(x_8 - x_4^2)}},$$

$$g_3(x_1, x_2, ..., x_{11}) = \frac{x_{11} - x_3x_4}{\sqrt{(x_7 - x_3^2)(x_8 - x_4^2)}}.$$

Then, $r_1 = g_1(\bar{\mathbf{Z}})$, $r_2 = g_2(\bar{\mathbf{Z}})$ and $r_3 = g_3(\bar{\mathbf{Z}})$.
By the delta method,

$$\sqrt{n}(r_1 - \beta, r_2 - \beta\rho_1, r_3 - \beta\rho_2) \xrightarrow{d} N(\mathbf{0}, \Gamma),$$

where $\Gamma_{ij} = \sum_{k=1}^{11}\sum_{l=1}^{11} \sigma_{kl} \frac{\partial g_i}{\partial \mu_k}\frac{\partial g_j}{\partial \mu_l}; i = 1,2,3; j = 1,2,3.$
Now,

$$\frac{\partial g_1}{\partial \mu_1} = \frac{\partial g_1}{\partial \mu_2} = \frac{\partial g_1}{\partial \mu_3} = \frac{\partial g_1}{\partial \mu_4} = \frac{\partial g_1}{\partial \mu_6} = \frac{\partial g_1}{\partial \mu_7} = \frac{\partial g_1}{\partial \mu_{10}} = \frac{\partial g_1}{\partial \mu_{11}} = 0,$$

$$\frac{\partial g_1}{\partial \mu_5} = \frac{\partial g_1}{\partial \mu_8} = -\frac{1}{2}\beta, \ \frac{\partial g_1}{\partial \mu_9} = 1.$$

$$\frac{\partial g_2}{\partial \mu_1} = \frac{\partial g_2}{\partial \mu_2} = \frac{\partial g_2}{\partial \mu_3} = \frac{\partial g_2}{\partial \mu_4} = \frac{\partial g_2}{\partial \mu_5} = \frac{\partial g_2}{\partial \mu_7} = \frac{\partial g_2}{\partial \mu_9} = \frac{\partial g_2}{\partial \mu_{11}} = 0,$$
$$\frac{\partial g_2}{\partial \mu_6} = \frac{\partial g_2}{\partial \mu_8} = -\frac{1}{2}\beta\rho_1, \ \frac{\partial g_2}{\partial \mu_{10}} = 1.$$

$$\frac{\partial g_3}{\partial \mu_1} = \frac{\partial g_3}{\partial \mu_2} = \frac{\partial g_3}{\partial \mu_3} = \frac{\partial g_3}{\partial \mu_4} = \frac{\partial g_3}{\partial \mu_5} = \frac{\partial g_3}{\partial \mu_6} = \frac{\partial g_3}{\partial \mu_9} = \frac{\partial g_3}{\partial \mu_{10}} = 0,$$
$$\frac{\partial g_3}{\partial \mu_7} = \frac{\partial g_3}{\partial \mu_8} = -\frac{1}{2}\beta\rho_2, \ \frac{\partial g_3}{\partial \mu_{11}} = 1.$$

Since the partial derivative matrix is very sparse, we don't need to calculate all the terms of the matrix Σ. The ones that are needed are calculated below.
$\sigma_{5,6} = E(X_1^2X_2^2) - 1 = 2\rho_1^2 + 1 - 1 = 2\rho_1^2$
$\sigma_{5,8} = E(X_1^2Y^2) - 1 = 2\beta^2 + 1 - 1 = 2\beta^2$
$\sigma_{5,10} = E(X_1^2X_2Y) - \beta\rho_1 = 3\beta\rho_1 - \beta\rho_1 = 2\beta\rho_1$
$\sigma_{8,6} = E(X_2^2Y^2) - 1 = 2\beta^2\rho_1^2 + 1 - 1 = 2\beta^2\rho_1^2$
$\sigma_{8,8} = E(Y^4) - 1 = 2$
$\sigma_{8,10} = E(X_2Y^3) - \beta\rho_1 = 3\beta\rho_1 - \beta\rho_1 = 2\beta\rho_1$
$\sigma_{9,6} = E(X_1X_2^2Y) - \beta = 2\beta\rho_1^2 + \beta - \beta = 2\beta\rho_1^2$
$\sigma_{9,8} = E(X_1Y^3) - \beta = 3\beta - \beta = 2\beta$
$\sigma_{9,10} = E(X_1X_2Y^2) - \beta^2\rho_1 = 2\beta^2\rho_1 + \rho_1 - \beta^2\rho_1 = \rho_1(1 + \beta^2)$
$\sigma_{6,7} = E(X_2^2X_3^2) - 1 = 2\rho_3^2 + 1 - 1 = 2\rho_3^2$
$\sigma_{6,11} = E(X_2^2X_3Y) - \beta\rho_2 = 2\beta\rho_1\rho_3 + \beta\rho_2 - \beta\rho_2 = 2\beta\rho_1\rho_3$
$\sigma_{8,7} = E(X_3^2Y^2) - 1 = 2\beta^2\rho_2^2 + 1 - 1 = 2\beta^2\rho_2^2$
$\sigma_{8,11} = E(X_3Y^3) - \beta\rho_2 = 3\beta\rho_2 - \beta\rho_2 = 2\beta\rho_2$
$\sigma_{10,7} = E(X_2X_3^2Y) - \beta\rho_2 = 2\beta\rho_2\rho_3 + \beta\rho_2 - \beta\rho_2 = 2\beta\rho_2\rho_3$
$\sigma_{10,11} = E(X_2X_3Y^2) - \beta^2\rho_1\rho_2 = \rho_3 + 2\beta^2\rho_1\rho_2 - \beta^2\rho_1\rho_2 = \rho_3 + \beta^2\rho_1\rho_2$
Combining, we get,

$$Cov(\sqrt{n}(r_1 - \beta), \sqrt{n}(r_2 - \beta\rho_1)) = \frac{\rho_1}{2}(1 - \beta^2)(2 - \beta^2 - \beta^2\rho_1^2),$$

$$Cov(\sqrt{n}(r_2 - \beta\rho_1), \sqrt{n}(r_3 - \beta\rho_2)) = 2\rho 3 + \beta^2(\rho_1^2 + \rho_2^2)(\beta^2\rho_1\rho_2 - 2\rho 3) + \beta^2\rho_1\rho_2(\rho_3^2 - 1).$$

Also,

$$Var(\sqrt{n}(r_1 - \beta)) = (1 - \beta^2)^2, \ Var(\sqrt{n}(r_2 - \beta\rho_1)) = (1 - \beta^2\rho_1^2)^2, \ Var(\sqrt{n}(r_3 - \beta\rho_2)) = (1 - \beta^2\rho_2^2)^2.$$

Hence,

$$Cor(r_1, r_2) = \rho_{12} = \frac{\rho_1(2 - \beta^2 - \beta^2\rho_1^2)}{2n(1 - \beta^2\rho_1^2)}$$

and

$$Cor(r_2, r_3) = \rho_{23} = \frac{2\rho 3 + \beta^2(\rho_1^2 + \rho_2^2)(\beta^2\rho_1\rho_2 - 2\rho 3) + \beta^2\rho_1\rho_2(\rho_3^2 - 1)}{2n(1 - \beta^2\rho_1^2)(1 - \beta^2\rho_2^2)}.$$

□

Corollary 1.1. *Let z_1, z_2 and z_3 be the Fisher transformed unscaled z-statistics corresponding to r_1, r_2 and r_3. Then,*

$$\sqrt{n-3}\begin{pmatrix} z_1 - tanh^{-1}(\beta) \\ z_2 - tanh^{-1}(\beta\rho_1) \\ z_3 - tanh^{-1}(\beta\rho_2) \end{pmatrix} \xrightarrow{d} N(\mathbf{0}, \begin{bmatrix} 1 & \rho_{12} & \rho_{13} \\ \rho_{12} & 1 & \rho 23 \\ \rho_{13} & \rho_{23} & 1 \end{bmatrix}),$$

where

$$\rho_{12} = \frac{\rho_1(2 - \beta^2 - \beta^2\rho_1^2)}{2(1 - \beta^2\rho_1^2)}$$

and

$$\rho_{23} = \frac{2\rho 3 + \beta^2(\rho_1^2 + \rho_2^2)(\beta^2\rho_1\rho_2 - 2\rho 3) + \beta^2\rho_1\rho_2(\rho_3^2 - 1)}{2(1 - \beta^2\rho_1^2)(1 - \beta^2\rho_2^2)},$$

ρ_{13} having the same form as ρ_{12}.

Corollary 1.2. *The covariance of the z-statistics converge to the covariance matrix for the case $\beta = 0$ as $|\rho_1| \to 1$ and $|\rho_2| \to 1$, or $|\rho_1| \to 0$ and $|\rho_2| \to 0$. This is also true for the conditional mean $E(z_2, z_3|z_1)$.*

The proof of Corollary 1.1 and Corollary 1.2 follows directly from Lemma 1. Clearly, similar results apply to more than three variables. Corollary 1.2 immediately implies that the conditional distribution of $(z_2, z_3|z_1)$ is approximately free of β when the correlations ρ_1 and ρ_2 are very large or very small. So, if the data has a block structure where there is very high correlation among SNPs within a block and there is very small correlation across blocks, then assumption (A3) may hold approximately, in a manner that supports the use of Z-REG-FDR.

To understand the difference between null and alternative of the conditional covariance matrices and mean vectors, we calculated the large sample means and covariance matrices under the two cases using Corollary 1.2 of Lemma 1. The dependence structure among the SNPs is (i) assumed to be an AR(1) structure with serial correlation 0.9, (ii) obtained from a real SNP matrix [1].

For case (i), Figure 1 shows the plot of the elements of the conditional covariance matrix under the null and that under the alternative for different effect sizes. The maximum

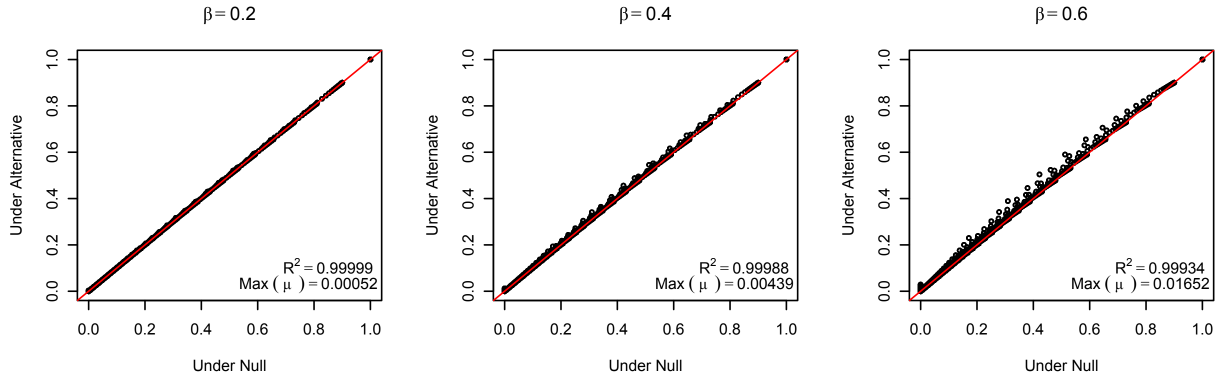

Supplementary Figure 1: Comparing the elements of conditional covariance matrix of Z under the null and those under the alternative. The R^2 as well as the maximum difference in the conditional means are reported. The correlation structure of the SNPs is assumed to be AR(1). β is the effect size.

difference in the conditional mean is also reported for each case. Figure 2 shows the same plot for case (ii). The fact that the differences are small, especially for the real SNP matrix, is an encouraging sign in favor of *Z-REG-FDR*. Figure 3 shows that under simulations, the estimated FDR based on true parameters agrees with estimated FDR based on *Z-REG-FDR* and *REG-FDR* both.

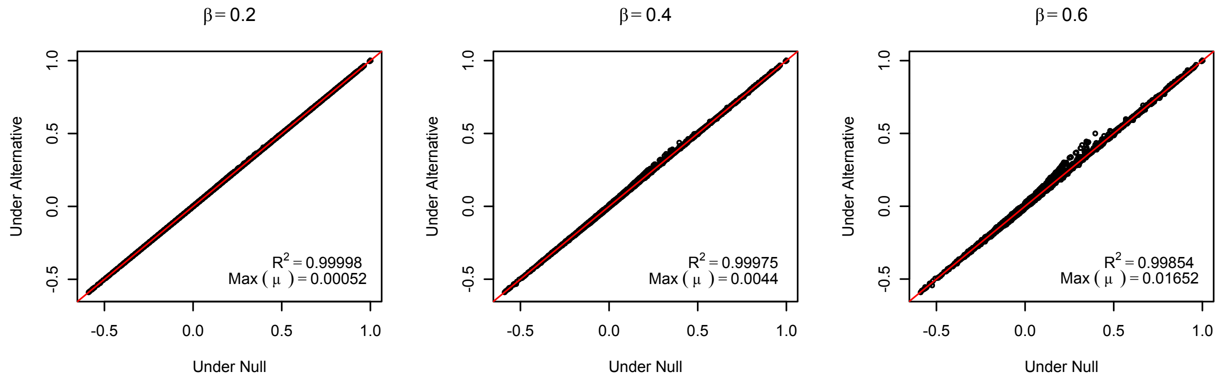

Supplementary Figure 2: Comparing the elements of conditional covariance matrix of Z under the null and those under the alternative. The R^2 as well as the maximum difference in the conditional means are reported. The correlation structure of the SNPs is obtained from a real data. β is the effect size.

3 Effect of more than one causal SNPs

One concern about our model is that it may have limited applicability for large cis-windows since it uses the assumption of only one causal SNP. We have explored through simulation the effect of more than one causal SNPs on the control of the FDR. We observed that under certain conditions, even in the presence of two causal SNPs, *Z-REG-FDR* is only very slightly anti-conservative.

True π_0	True σ	Mean $\hat{\pi_0}$	Mean $\hat{\sigma}$	SE($\hat{\pi_0}$)	SE($\hat{\sigma}$)	Realized FDR(5%)	Realized FDR(10%)
0.10	1	0.2178	1.1354	0.0800	0.0508	0.0320	0.0533
0.10	2	0.0942	2.1099	0.0237	0.0264	0.0566	0.0945
0.10	5	0.0884	5.1313	0.0070	0.0218	0.0574	0.0999
0.20	1	0.3039	1.1353	0.0764	0.0550	0.0439	0.0780
0.20	2	0.1926	2.1071	0.0241	0.0294	0.0545	0.1066
0.20	5	0.1885	5.1269	0.0077	0.0278	0.0549	0.1075

Supplementary Table 1: Showing summary of the simulation studies for two causal SNPs

Table 1 shows the results for simulated dataset. Under the alternative hypothesis, the expressions are simulated using one primary causal SNP for which the Fisher transformed effect size follows a normal distribution with standard deviation σ, and there might exist (with probability 1/2) a secondary causal SNP which has an effect size that is smaller in magnitude and has the same sign as the primary effect size. Note that it is not possible to have the secondary effect size to be unconstrained and at the same time maintain the desired variance of Y. It can be shown that the simulation using the above mentioned conditions is always feasible. Table 1 demonstrates that if the secondary effect size is not very large and has the same direction, then *Z-REG-FDR* achieves reasonable control of the FDR.

4 Additional Supplementary Figures and Tables

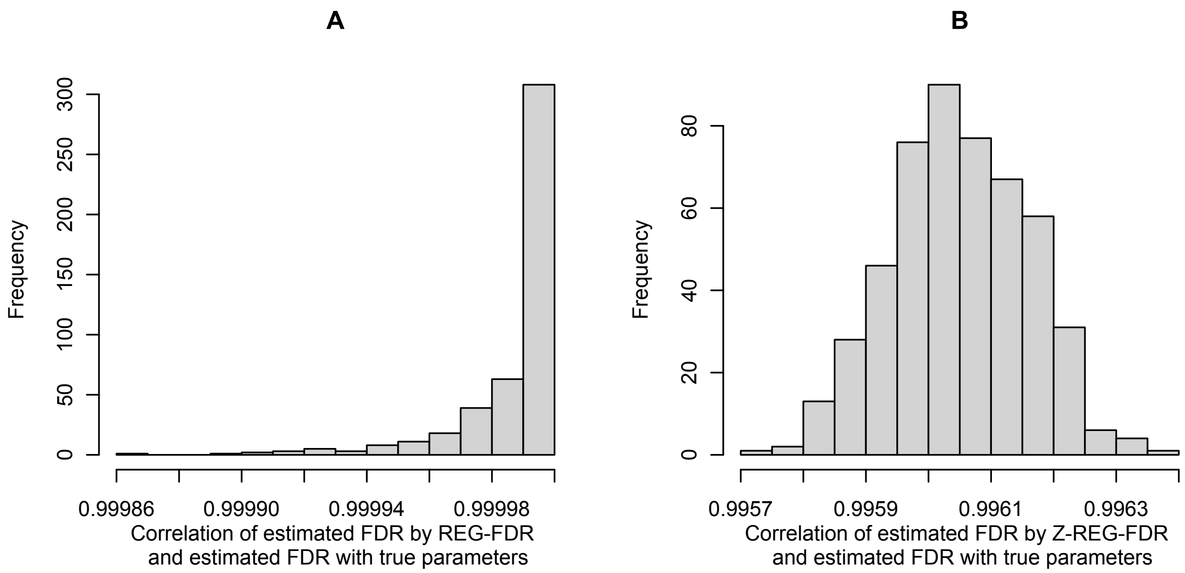

Supplementary Figure 3: Showing the histograms of correlations between the estimated FDR based on the true values of the parameters and that based on **A.** *REG-FDR* **B.** *Z-REG-FDR*. Simulation was conducted using the scheme described in Section 3.2 in the main paper.

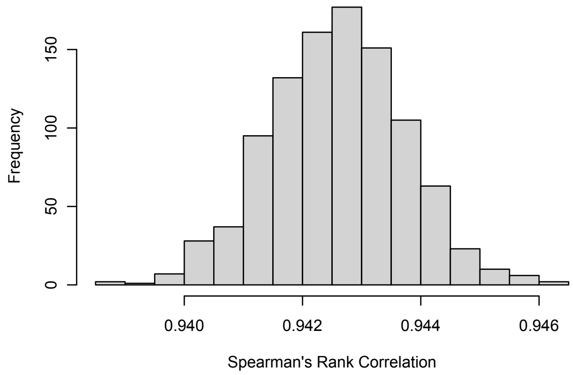

Supplementary Figure 4: Showing the histogram of correlations between estimated FDR using the permutation method and that using *Z-REG-FDR*.

		Number of significant genes		
Tissue	n	*Z-REG-FDR*	Simes	Permutation
Subcutaneous adipose	298	7995	6963	6604
Visceral omentum	185	4231	3571	3501
Adrenal gland	126	2866	2693	2514
Aorta	197	5150	5162	4487
Coronary artery	118	2032	1882	1822
Tibial artery	285	7729	6736	6368
Anterior cingulate cortex BA24	72	1145	938	1044
Caudate nucleus	100	1945	1967	1796
Cerebellar hemisphere	89	3500	2557	2705
Cerebellum	103	3560	3454	3117
Cortex	96	2031	2086	1889
Frontal cortex (BA9)	92	1514	1588	1436
Hippocampus	81	1046	853	942
Hypothalamus	81	1113	879	1014
Nucleus accumbens (basal ganglia)	93	1554	1617	1445
Putamen (basal ganglia)	82	1530	1238	1310
Breast mammary tissue	183	4019	3271	3421
EBV-transformed lymphocytes	114	2558	2360	2287
Fibroblasts	272	8678	7513	6947
Sigmoid colon	124	2544	2269	2258
Transverse colon	169	4406	3723	3662
Gastroesophageal junction	127	2489	2237	2225
Esophagus mucosa	241	6794	6169	5700
Esophagus muscularis	218	6126	5731	5234
Atrial appendage	159	3746	3284	3137
Left ventricle	190	4484	3855	3716
Liver	97	1242	1231	1184
Lung	278	6815	5884	5818
Skeletal muscle	361	7175	6049	5841
Tibial nerve	256	9374	8087	7640
Ovary	85	1404	1167	1259
Pancreas	149	3938	3621	3352
Pituitary	87	2168	1607	1861
Prostate	87	1391	1045	1233
Skin (Not sun-exposed)	196	4373	4499	3905
Skin (Sun-exposed)	302	8304	7109	6882
Small intestine terminal ileum	77	1306	1002	1150
Spleen	89	2822	2163	2267
Stomach	170	3420	2938	2927
Testis	157	8430	6796	7003
Thyroid	278	9498	7976	7809
Uterus	70	882	655	774
Vagina	79	933	582	792
Whole blood	338	6887	5862	5814

Supplementary Table 2: Number of significant genes found by different methods across the tissues of the GTEx data

References

[1] John Lonsdale, Jeffrey Thomas, Mike Salvatore, Rebecca Phillips, Edmund Lo, Saboor Shad, Richard Hasz, Gary Walters, Fernando Garcia, Nancy Young, et al. The genotype-tissue expression (gtex) project. *Nature genetics*, 45(6):580–585, 2013.
